# A refined model of the genomic basis for phenotypic variation in vertebrate hemostasis

**DOI:** 10.1186/s12862-015-0409-y

**Published:** 2015-06-30

**Authors:** Ângela M. Ribeiro, M. Lisandra Zepeda-Mendoza, Mads F. Bertelsen, Annemarie T. Kristensen, Erich D. Jarvis, M. Thomas P. Gilbert, Rute R. da Fonseca

**Affiliations:** Interdisciplinary Centre of Marine and Environmental Research—CIIMAR/CIMAR, University of Porto, Rua dos Bragas 289, 4050-123 Porto, Portugal; Centre for GeoGenetics, Natural History Museum of Denmark, University of Copenhagen, Øster Voldgade 5-7, 1350 Copenhagen, Denmark; Centre for Zoo and Wild Animal Health, Copenhagen Zoo, Roskildevej 38, 2000 Frederiksberg, Denmark; Department of Veterinary Clinical and Animal Sciences, Faculty of Health and Medical Sciences, University of Copenhagen, DK-1870 Frederiksberg C, Denmark; Department of Neurobiology, Duke University Medical Centre, Durham, NC 27710 USA; Howard Hughes Medical Institute, Chevy Chase, MD 20815 USA; Trace and Environmental DNA Laboratory, Department of Environment and Agriculture, Curtin University, Perth, WA 6102 Australia; The Bioinformatics Centre, University of Copenhagen, Copenhagen, Denmark

## Abstract

**Background:**

Hemostasis is a defense mechanism that enhances an organism’s survival by minimizing blood loss upon vascular injury. In vertebrates, hemostasis has been evolving with the cardio-vascular and hemodynamic systems over the last 450 million years. Birds and mammals have very similar vascular and hemodynamic systems, thus the mechanism that blocks ruptures in the vasculature is expected to be the same. However, the speed of the process varies across vertebrates, and is particularly slow for birds. Understanding the differences in the hemostasis pathway between birds and mammals, and placing them in perspective to other vertebrates may provide clues to the genetic contribution to variation in blood clotting phenotype in vertebrates. We compiled genomic data corresponding to key elements involved in hemostasis across vertebrates to investigate its genetic basis and understand how it affects fitness.

**Results:**

We found that: i) fewer genes are involved in hemostasis in birds compared to mammals; and ii) the largest differences concern platelet membrane receptors and components from the kallikrein-kinin system. We propose that lack of the cytoplasmic domain of the GPIb receptor subunit alpha could be a strong contributor to the prolonged bleeding phenotype in birds. Combined analysis of laboratory assessments of avian hemostasis with the first avian phylogeny based on genomic-scale data revealed that differences in hemostasis within birds are not explained by phylogenetic relationships, but more so by genetic variation underlying components of the hemostatic process, suggestive of natural selection.

**Conclusions:**

This work adds to our understanding of the evolution of hemostasis in vertebrates. The overlap with the inflammation, complement and renin-angiotensin (blood pressure regulation) pathways is a potential driver of rapid molecular evolution in the hemostasis network. Comparisons between avian species and mammals allowed us to hypothesize that the observed mammalian innovations might have contributed to the diversification of mammals that give birth to live young.

**Electronic supplementary material:**

The online version of this article (doi:10.1186/s12862-015-0409-y) contains supplementary material, which is available to authorized users.

## Background

Vertebrates possess a striking ability to minimize blood loss after tissue injury. This defense mechanism is called hemostasis and implies a delicate equilibrium between coagulation with formation of a fibrin-platelet mesh and fibrinolysis. The importance of this equilibrium is evident in placental mammals, where the separation of the highly vascularized placenta poses a challenge to hemostasis, such that late pregnancy is characterized by a hypercoagulability status: increased concentration of clotting factors and a decrease in the amount of anticoagulants in the blood [1]. A tight regulation of hemostasis is also crucial for deep-diving vertebrates such as cetaceans (mammals), penguins (birds) and turtles (reptiles). During diving, blood is primarily directed into the oxygen-dependent brain while the flow into visceral organs becomes sluggish, thus increasing the risk of clotting [2]. A fine-tuning of hemostasis is also needed during the molting period in birds because damage to the highly irrigated growing feathers can prove fatal.

**Fig. 1:**
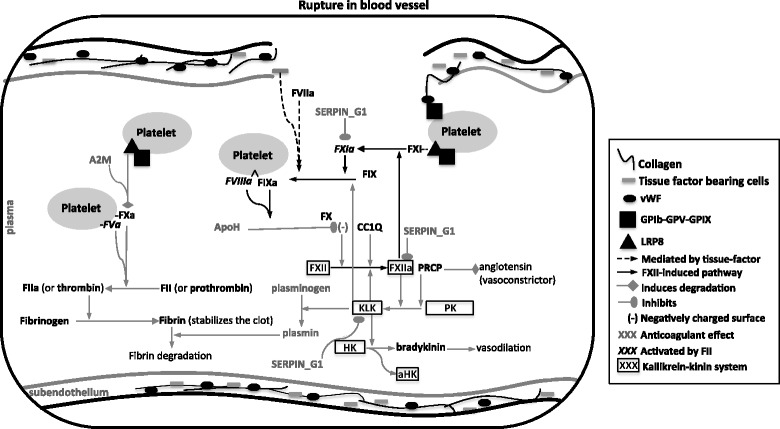
Cell-based model of hemostasis. Depiction of the hemostasis process according to the well-known human system. All the components (proteins) discussed in this study are included. Full names of the proteins are provided in Additional file 1: Table S1 (supplementary material)

The hemostatic mechanism involves three main components: i) vasculature/endothelial, ii) platelet/thrombocytes and iii) plasma proteins. At the physiological level, hemostasis is responsible for the production of a hemostatic plug to impede exsanguination after vascular injury. As determined in well-studied mammalian systems [3] damage to the blood vessel wall exposes the sub-endothelia layer and the embedded tissue-factor-bearing cells, triggering thrombin formation and subsequent platelet mobilization to the site of injury and the formation of a stable fibrin clot (Fig. 1; detailed description on Additional file 1: Supplementary Material).

The current understanding of the biochemical processes of hemostasis was contributed by extensive studies in the context of human disease (e.g., [4–6]) and few other model systems (e.g. [7]). It is now established that hemostasis results from the action of an intricate network of molecules [2], which increased in complexity throughout vertebrate evolution, over the last 450 million years [8]. This complexity has been linked to the evolution of closed and highly pressurized circulatory systems and appearance of novel organs such as lungs, and subsequent need to respond to trauma in a vessel wall more rapidly. Moreover, the refinement of the hemostasis in mammals has been proposed to result from overlaps with the inflammation pathway [9] and the complement pathway [10]. This tight relationship between inflammation, complement, and coagulation was confirmed by recent work at the genomic level [11, 12], and has shaped our understanding of the repertoire and features of the components of hemostasis in mammals relative to other vertebrate groups. The major difference among vertebrates pertains to the plasma kallikrein-kinin system: FXII, paralogs FXI and prekallikrein, and kininogen [13]. FXII is absent in fish, birds [8] and pseudogeneized in Cetaceans [14]. FXI and plasma kallikrein result from a gene duplication that occurred in the mammalian lineage [14]. Complexity in the structure of kininogen increased throughout the evolution of vertebrates via shuffling of protein domains [9].

Similarities between mammal and bird vascular and hemodynamic systems include that they have the highest arterial blood pressures of all vertebrates [15]. Therefore, the need to rapidly seal the circulatory system in case of rupture is expected to be similar. In this regard, platelets and thrombocytes in mammals and birds, respectively, respond to the same primary agonists, for instance exposed collagen fibers of the vessel wall [16]. Nevertheless, birds have more prolonged bleeding upon vascular injury (e.g., [17, 18]). While in mammals platelets rapidly form three-dimensional aggregates, in birds their thrombocytes adhere and spread, but do not form aggregates. Rather, they have a monolayer of a single thrombocyte with a surface area 9–12 times larger that takes longer to expand and cover the damaged area [16].

The sturdiness of the clot is a variable feature within birds [19]. Together these features make birds an outstanding group to understand the effect that genetic differences within the hemostasis pathway have on the blood clotting phenotype in vertebrates, and suggest the platelet component of hemostasis might be the key to understanding the differences in hemostatic response between mammals and birds.

In this study, we explored a large collection of available genomic and transcriptomic data across vertebrates to re-assess the differences in proteins from the core of the hemostatic pathway. We compared a set of five representative mammals with 48 bird species across the avian phylogeny provided by our companion study [20], 12 reptile, three amphibian and three fish species (Additional file 1: Table S2). This provided a more comprehensive overview of the conservation of hemostasis genes along the vertebrate tree, and led us to revise our understanding of the evolutionary advantages of observed differences between groups. We also confirmed that the hemostasis network in birds is more similar to other archosaurs and the turtle sister taxa, despite the similarity between the avian and mammalian hemostatic needs. Finally, using the first genomic-scale avian phylogeny as a base [21], we found significant associations between the clot strength within birds and the genetic distance between genes that are involved in hemostasis.

## Results and discussion

### Molecular differences in hemostasis components across vertebrates

**Fig. 2:**
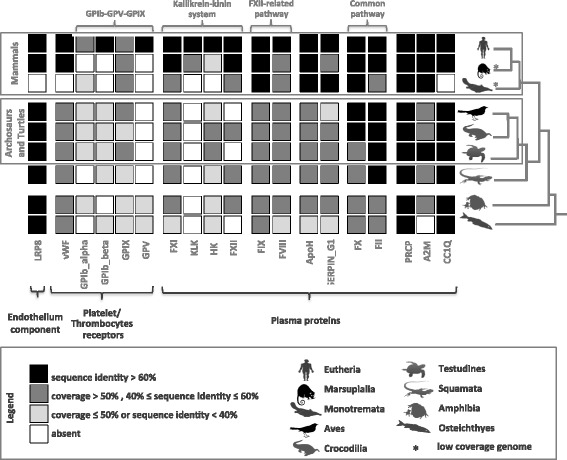
Orthologs of genes involved in hemostasis across vertebrates. Repertoire of putative orthologs across vertebrates of human genes involved in hemostasis. The color scheme summarizes two parameters: coverage (portion of the sequence aligned to the human reference) and protein sequence identity

Our analysis of genomic-level data confirmed [4] that mammals have more hemostasis-related genes than the other vertebrates (Fig. 2). However, we found that the main differences are within the platelet component of the network, i.e., in the platelet membrane receptors (Fig. 2 and Additional file 1: Figure S1).

### The platelet receptor GPIb-V-IX

In the mammalian model of hemostasis [3] platelets are mobilized to the site of injury after an initial burst of thrombin to initiate the formation of a plug that impedes exsanguination. Avian thrombocytes have been thought to function similarly [13], but this has not been assessed at the molecular level. We found that the platelet receptor GPIb*alpha* had less than 50 % of the sequence present (Cov. < 50 %) in all vertebrates except placental mammals (Fig. 2). A detailed inspection of the sequences revealed that the cytoplasmic domains, which is responsible for inducing the intracellular signal necessary for shape change and adhesion in mammalian platelets, was lacking in archosaur (birds and crocodiles) and turtle species examined (Additional file 1: Figure S2; but see [22] for an detailed illustration of GPIb complex and the putative location of the region mentioned).

We interpret this finding to suggest that if, as in mammalian platelets [21], avian thrombocytes bind to the subendothelial-deposited vWF through the extra-cellular domain of GP1b*alpha*, then the lack of the cytoplasmic domain indicates that cytoskeletal rearrangements required for platelet shape might be affected. This feature of GPIb*alpha* might help to explain why, unlike mammalian platelets, avian thrombocytes take longer to produce a clot to control bleeding, as well as explain the previous finding that avian thrombocytes cannot form tight aggregates [16].

Our findings also support that a fully functional platelet receptor GPIb-V-IX (comprising GPIb*alpha*, GPIb*beta*, GPIX, GPV) appears to be a placental mammalian innovation (Fig. 2) likely to boost thrombin formation and the activation of platelets. The production of thrombin is triggered not only through the formation of the pro-thrombinase complex but also when Factor XI (FXI) binds to GPIb*alpha* and/or LRP8 [23]. In contrast to GPIb*alpha*, we found LRP8 to be ubiquitous in vertebrates. Therefore, the incomplete GPIb*alpha* in birds suggests that the action of FXI, which we find in all birds (Fig. 2), might be reduced to LRP8, possibly affecting thrombin production (quantitatively) and the time to efficient clot formation. The mammalian innovations in the molecular machinery of platelets provide an ability to form efficient clots rapidly. We hypothesize this novelty in platelet’s receptors, together with development of FXII and plasma kallikrein related systems, might have contributed to the radiation of mammals that give birth to live young (therians) because it provides an efficient mechanism for fast and effective control of small ruptures at the placenta during pregnancy, as well as to prevent fatal bleeding from the placental site after delivery.

### Factor XII (Hageman factor), HK and FXI-KLK

Our genomic data supports [8, 14] absence of FXII in birds and we add that it is likely part of a eutherian mammalian innovation because we only found non-mammalian BLAST hits with very low protein sequence identities with mammalian ones, and therefore unlikely to have the same function as in mammals (Fig. 2 and Additional file 1: Figure S4). We confirm the suggestion that FXI and KLK are the result of a duplication that occurred in the therian lineage, because in the prototheria platypus, as in all other vertebrates, FXI-KLK exists as a single gene (Additional file 1: Figure S3).

A puzzling result pertains the low coverage in HK in all but eutherian mammals. This gene encodes the high molecular weight kininogen (HK) and low molecular weight kininogen, proteins that belong to the hemostasis and inflammation processes, respectively and thus central to control the response to tissue damage and infection. Production of thrombin via FXII and release of bradykinin after HK cleavage by plasma kallikrein is triggered by the exposure to negatively charged surfaces, such as bacterial cell walls [24]. The aim is to impede the spread of an infection through blood flow by blocking small veins with clots. Concomitant to the low coverage in HK in all but eutherian mammals, we found absence of plasma kallikrein in non-mammals and monotremata (Fig. 2). This indicates that the evolution of the kallikrein-kinin system is a eutherian mammal innovation, which we suggest might help fight the spread of infection to the fetus via the placenta. This is consistent with Cagliani *et al*. [25] who suggested that HK has been evolving adaptively in mammals, under balancing selection.

### Phenotypic divergence within birds

**Fig. 3:**
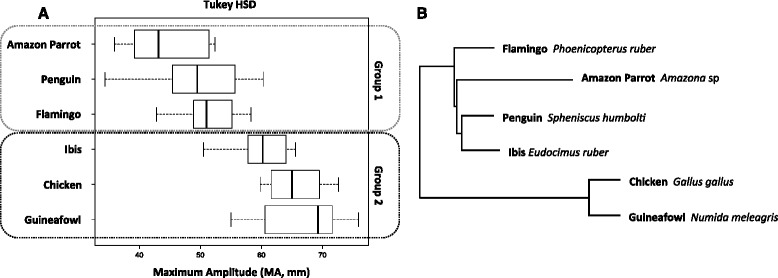
Coagulation phenotype divergence among the six avian lineages analyzed. **a** Two groups statistically supported for clot Maximum Amplitude (MA); **b** phylogeny used to account for shared ancestry in the Phy-ANOVA

Studies on functionality of hemostasis within birds are scarce [18, 26] possibly because birds have cardiovascular physiologies that closely resemble mammals and hence sorely overlooked. We found interspecific differences of clot strength among birds (Fig. 3a; ANOVA: F_5,64_ = 26.126, *p* < 0.001). When controlling for the possible effects of phylogenetic relationships (Fig. 3b), chicken, Guinea fowl and ibis produce clots that are significantly more resistant than in flamingo, penguin and parrot (Fig. 3a; Phy-ANOVA: F_1,4_ = 26.542, *p* < 0.05). These differences in clot strength due to platelet function were associated with evolutionary changes at two plasma proteins as measured with Jones-Taylor-Thorton amino acid distances (Table 1): thrombin (FII) and prolylcarboxypeptidase (PRCP).

**Table 1:** Mantel and partial Mantel’s tests of physiological genetics (MA) and phylogenetic distance matrices

	Functional component	Variable	R	p
Physiological genetic model	Endothelium	LRP8	0.589	0.021
	Platelets/Thrombocytes	GPIX	0.067	0.354
	Plasma Proteins	ApoH	0.689	0.017
		CC1Q	0.589	0.014
		FII	0.727	0.009
		PRCP	0.707	0.007
Physiological genetic model with phylogeny	Endothelium	LRP8	0.282	0.136
	Platelets/Thrombocytes	GPIX	−0.017	0.464
	Plasma Proteins	ApoH	0.489	0.027
		CC1Q	0.355	0.073
		**FII**	0.569	**0.008**
		**PRCP**	0.530	**0.011**

The regression coefficient (R) and associated p-values (p) are shown for i) the physiological genetic model, showing the results from Mantel tests for each variable, and ii) the physiological genetic model with phylogeny shows the results from partial Mantel tests controlling for phylogenetic distances (significant variables after correcting for FDR are highlighted in bold)

Plasma protein PRCP is not recognized as a hemostatic protein. Its function includes regulation of blood pressure and thrombosis risk, and modulation of vascular growth and repair after injuries [26]. Regarding blood pressure, PRCP action counterbalances the vasoconstrictive effects of angiotensin II and leads to blood pressure reduction via bradykinin [27]. Relating to hemostasis, in mammals, PRCP participates, indirectly, in the activation of hemostatic protein FXII through a mechanism that involves the activation of prekallikrein into plasma kallikrein [28]. The apparent relevance of PRCP in clot strength in birds is striking due to the lack of FXII and plasma kallikrein. This result warrants further investigation to disentangle potential co-evolution with other hemostatic proteins.

## Conclusions

Mapping genotype to phenotype is considered one of the ‘Grand Challenges’ of 21st Century Biology [29]. This is a first attempt across many vertebrate genomes to start addressing questions about the evolution of hemostasis by combining genomic and phenotypic data. In physiological processes composed of many elements, phenotypes might be dictated by the interactions between multiple components (e.g., [30]); this might be the case for hemostasis. Synergistic effects between elements of hemostasis and elements from the complement pathway as part of innate immunity (e.g., [10]) might influence an individual’s fitness and hence are targets of natural selection.

Here we establish that hemostasis in birds involves fewer genes than in mammals. We also demonstrate that within birds, at the inter-specific level, the efficiency to produce a plug is best explained by genetic variation in key components of the hemostatic process than by phylogenetic relationships. Our study opens ground for future work in several fields: for human-based research, by informing when the molecular machinery underlying hemostasis was established; for avian physiology research and veterinary medicine, by increasing our understanding of genes involved in avian bleeding that will be useful for understanding bleeding disorders in captive and wild populations.

## Methods

### Orthology assignment across vertebrates for coagulation-related genes

The sequences of genes for 18 human proteins associated with platelets, the subendothelium layer, and plasma proteins participating in hemostasis (Additional file 1: Table S1), were recovered from ENSEMBLE v67 (http://www.ensembl.org). We used the longest transcript as probes in BLASTp and tBLASTn searches (e-value threshold of 10^−6^) against genome, transcriptome, and proteome data from species representing the vertebrate tree of life (Additional file 1: Table S2): fishes, amphibians, reptiles, birds and mammals.

An *all*-*vs*-*all* BLASTp of the 25,752 protein coding sequences in the dataset was applied and OrthoMCL [31] used to determine ortholog groups. We then used the R package *phangorn* [32] to compute amino-acid distances according to the WAG matrix [33], and performed data clustering using *bios2mds* [34]. To obtain the final alignment as implemented in PRANK [35], an algorithm that is phylogeny-aware, we first computed a guide phylogenetic tree with MAFTT v6.951b [36]. With the alignments we estimated coverage and protein percent identity of the top scoring coding regions per vertebrate group relative to the human sequences.

### Assessing the differences in avian hemostatic phenotypes

In order to assess the efficiency of hemostasis within birds, we used recently published data from a laboratory evaluation of whole blood clot formation [19]. In that study, evaluation of hemostasis was assessed by thromboelastography (TEG), a methodology widely used in mammals [37] and now validated for birds [19]. The TEG hemostatic assay measures the visco-elastic properties of whole blood clot formation. With the TEG results, we measured the maximum strength of the developed clot: Maximum Amplitude (MA, mm). This parameter depends on the concentration of fibrinogen and platelets/thrombocytes, as well as their function. The MA values in [19] correspond to six lineages representing five avian orders as defined in [21]: Galliformes (domestic chicken, *Gallus gallus*; helmeted guineafowl, *Numida meleagris*), Phoenicopteriformes (American flamingo, *Phoenicopterus ruber*), Sphenisciformes (Humboldt penguin, *Spheniscus humbolti*), Pelecaniformes (scarlet ibis, *Eudocimus ruber*) and Psittaciformes (Amazon parrot, *Amazona sp*.). We calculated the phenotypic distance between lineages as the Euclidean distance between the corresponding mean values in MA.

With the MA values, we characterize the differences in hemostatic phenotypes within birds by first testing for differences in maximum strength of the developed clot within birds using an ANOVA framework followed by multiple comparisons performed with the post-hoc Tukey’s HSD, after confirming normality with Shapiro’s test (Fig. 3a). We subsequently performed a phylogenetic ANOVA (Phy-ANOVA) to check if there were significant differences after accounting for the expected variation given the phylogenetic relationships (R Geiger package; [38]). For the Phy-ANOVA, we pruned an avian phylogeny [21] to include only the lineages for which we had MA estimates.

We then compared the phenotypes represented by MA values to the genetic distances between the genes underlying the hemostasis process within birds. We restricted our analysis to genes that: i) align > 50 % to the human ortholog; ii) are represented in the six avian lineages; and iii) show no duplication event within birds, to elude paralogy. We tested whether a similar MA value corresponds to a protein sequence similarity by conducting Mantel tests. We used a partial Mantel’s test to control for phylogenetic proximity, i.e., remove the phylogenetic signal while still testing the correlation between the first two matrices (phenotypic and genetic distances). We used the relative position of each lineage within the avian phylogeny to establish phylogenetic proximity (Table 2), and Jones-Taylor-Thornton matrices of amino acid substitutions [39] to estimate functional genetic distances. We corrected for false discovery rate in multiple comparisons using B-H’s method [40]. All statistical analyses were performed in the R Ecodist package ([41]; R Development Core Team 2014); Significance was calculated using 9999 random permutations of matrices.

**Table 2:** Phylogenetic relationships

Lineage1	Lineage2	Phylogenetic distance
Chicken	Amazon Parrot	5
*(Gallus gallus)*	*(Amazona sp.)*	
Guineafowl	Chicken	1
*(Numida meleagris)*	*(Gallus gallus)*	
Guineafowl	Amazon Parrot	5
*(Numida meleagris)*	*(Amazon sp)*	
Ibis	Chicken	4
*(Eudocimus ruber)*	*(Gallus gallus)*	
Ibis	Guineafowl	4
*(Eudocimus ruber)*	*(Numida meleagris)*	
Ibis	Amazon Parrot	2
*(Eudocimus ruber)*	*(Amazon sp)*	
Ibis	Flamingo	2
*(Eudocimus ruber)*	*(Phoenicopterus ruber)*	
Ibis	Penguin	1
*(Eudocimus ruber)*	*(Spheniscus humbolti)*	
Flamingo	Chicken	2
*(Phoenicopterus ruber)*	*(Gallus gallus)*	
Flamingo	Guineafowl	2
*(Phoenicopterus ruber)*	*(Numida meleagris)*	
Flamingo	Amazon Parrot	3
*(Phoenicopterus ruber)*	*(Amazon sp)*	
Penguin	Chicken	4
*(Spheniscus humbolti)*	*(Gallus gallus)*	
Penguin	Guineafowl	4
*(Spheniscus humbolti)*	*(Numida meleagris)*	
Penguin	Amazon Parrot	2
*(Spheniscus humbolti)*	*(Amazon sp)*	
Penguin	Flamingo	2
*(Spheniscus humbolti)*	*(Phoenicopterus ruber)*	

Conceptual phylogenetic distances between the six avian lineages based on the topology of the first genomic-level avian tree of life [21]. The pruned tree is depicted in Fig. 2

## Additional file

Additional file 1:
**Supplementary text, figures and tables.** Description of hemostasis, supplementary Figures 1 to 4 with more details on the orthologous relationships and supplementary Tables 1 and 2 with detailed information regarding genes, species and datasets.
