# Supplementary material for: A refined model of the genomic basis for phenotypic variation in vertebrate hemostasis
Source: BMC Evol Biol. 2015 Jun 30;15:124. doi: 10.1186/s12862-015-0409-y (PMC4486697; doi:10.1186/s12862-015-0409-y)
Supplement: Additional file 1: — Supplementary text, figures and tables. Description of hemostasis, supplementary Figures 1 to 4 with more details on the orthologous relationships and supplementary Tables 1 and 2 with detailed information regarding genes, species and datasets. [file 12862_2015_409_MOESM1_ESM.docx]

**SUPPLEMENTARY MATERIAL**

# A refined model of the genomic basis for phenotypic variation in vertebrate hemostasis

**Ângela M. Ribeiro^1^, M. Lisandra Zepeda-Mendoza^2^, Mads F. Bertelsen^3^, Annemarie T. Kristensen^4^, Erich D. Jarvis^5,6^, M. Thomas P. Gilbert^2,7^, Rute R. da Fonseca^1,2,8^**^§^

^1^ Interdisciplinary Centre of Marine and Environmental Research – CIIMAR/CIMAR, University of Porto, Rua dos Bragas 289, 4050-123 Porto, Portugal

^2^ Centre for GeoGenetics, Natural History Museum of Denmark, University of Copenhagen, Øster Voldgade 5-7, 1350, Copenhagen, Denmark

^3^ Centre for Zoo and Wild Animal Health, Copenhagen Zoo, Roskildevej 38, 2000 Frederiksberg, Denmark

^4^ Department of Veterinary Clinical and Animal Sciences, Faculty of Health and Medical Sciences, University of Copenhagen, DK-1870 Frederiksberg C, Denmark

^5^ Department of Neurobiology, Duke University Medical Centre, Durham, NC 27710, USA

^6^ Howard Hughes Medical Institute, Chevy Chase, MD 20815, USA

^7^ Trace and Environmental DNA Laboratory, Department of Environment and Agriculture, Curtin University, Perth, Western Australia, 6102, Australia

^8^ The Bioinformatics Centre, University of Copenhagen, Copenhagen, Denmark

^§^Corresponding author

Email addresses:

AMR: ribeiro.angela@gmail.com

MLZP: lisandracady@gmail.com

MFB: MFB@zoo.dk

ATK: atk@sund.ku.dk

ED: jarvis@neuro.duke.edu

MTPG: mtpgilbert@gmail.com

RRF: fonseca@binf.ku.dk

Supplementary Text

1.1. Main steps of the hemostasis network:

- The process of blood coagulation is initiated either by: a) the exposure of tissue factor and collagen or b) exposure of Factor XII (FXII) to negatively charged surfaces which results in the production of FXIIa (”a” denotes ”activated”).
- Tissue factor forms a complex with FVII and this activates FIX to FIXa.
- Factor XIIa then activates Factor XI, which in turn activates Factor IX that together with Factor VIIIa activates Factor X to Factor Xa (on the surface of platelets).
- Factor Xa in combination with Factor Va induces a thrombin (Factor II) burst, since a single FXa-FVa complex can activate many prothrombin molecules.
- The thrombin burst further mobilizes platelets to the site of the injury where they stick to collagen.
- Thrombin further activates FV, FVIII and FXI, in a positive feedback loop.
- Fibrinogen attaches itself to platelets.
- Thrombin promotes the formation of fibrin from fibrinogen.
- Fibrin stabilizes the platelet plug forming an arterial clot.
- Once the fibrin-platelet clot has formed, the clotting process is limited to avoid thrombotic occlusion of vasculature.

1.2. Other details:

- Platelets bind to the exposed subendothelium collagen in a ruptured blood vessel via alpha2beta1 integrin and GPVI.
- The binding at high shear occurs mainly through a complex formed by the glycoproteins Ib, V and IX (GPIb, GPV and GPIX), which cross-links with collagen-bound von Willebrand factor (vWF).
- The observed lack of thrombocyte-thrombocyte adhesion is explained by the density of α2bβ3 integrins (required for cell-cell adhesion) being at least 18-25 times lower in thrombocytes suggested to results from a lack of up-regulation of an ADP receptor (P2RY12) that regulates the α2bβ3 integrins during formation of shear-resistant platelet aggregates (avian thrombocytes do not respond strongly to ADP – see reference 16 in the main text).
- LRP8 (in a complex with GPIb) is the major recruiter of FXI to the platelet surface.
- FXI circulates in the plasma with KNG1.
- FVIII circulates in the plasma bound to vWF.
- FXI can be activated by FXIIa, FIIa and FVIIa.
- The tissue factor/Factor VIIa complex can also activate Factor IX.
- FIX recruits FVIIIa to activate FX an essential step in the intrinsic pathway.
- The adhesion of platelets to FXI is followed by platelet activation.
- LRP8, together with alpha-2-macroglobulin (A2M) contributes to the turnover of FXa.

1. Supplementary Figures

**
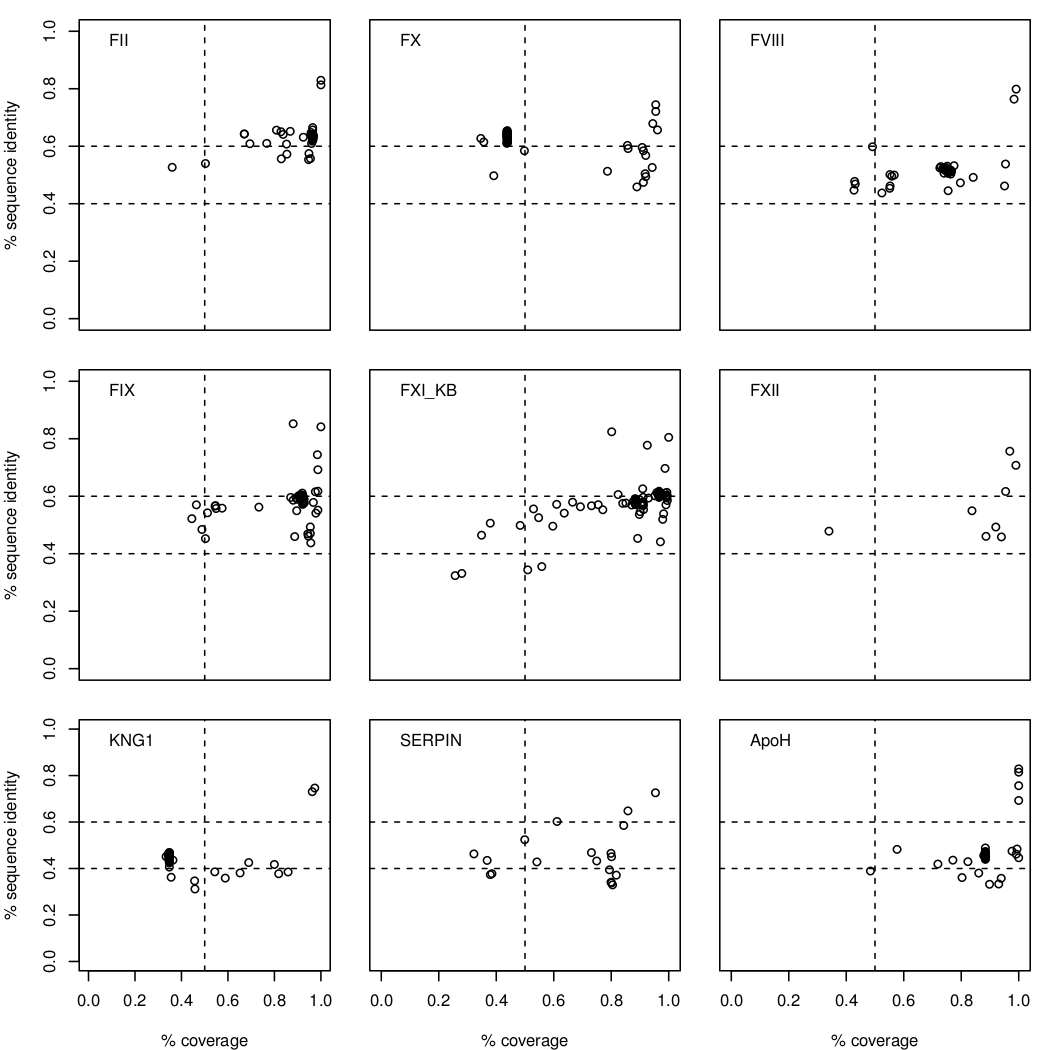
**

**
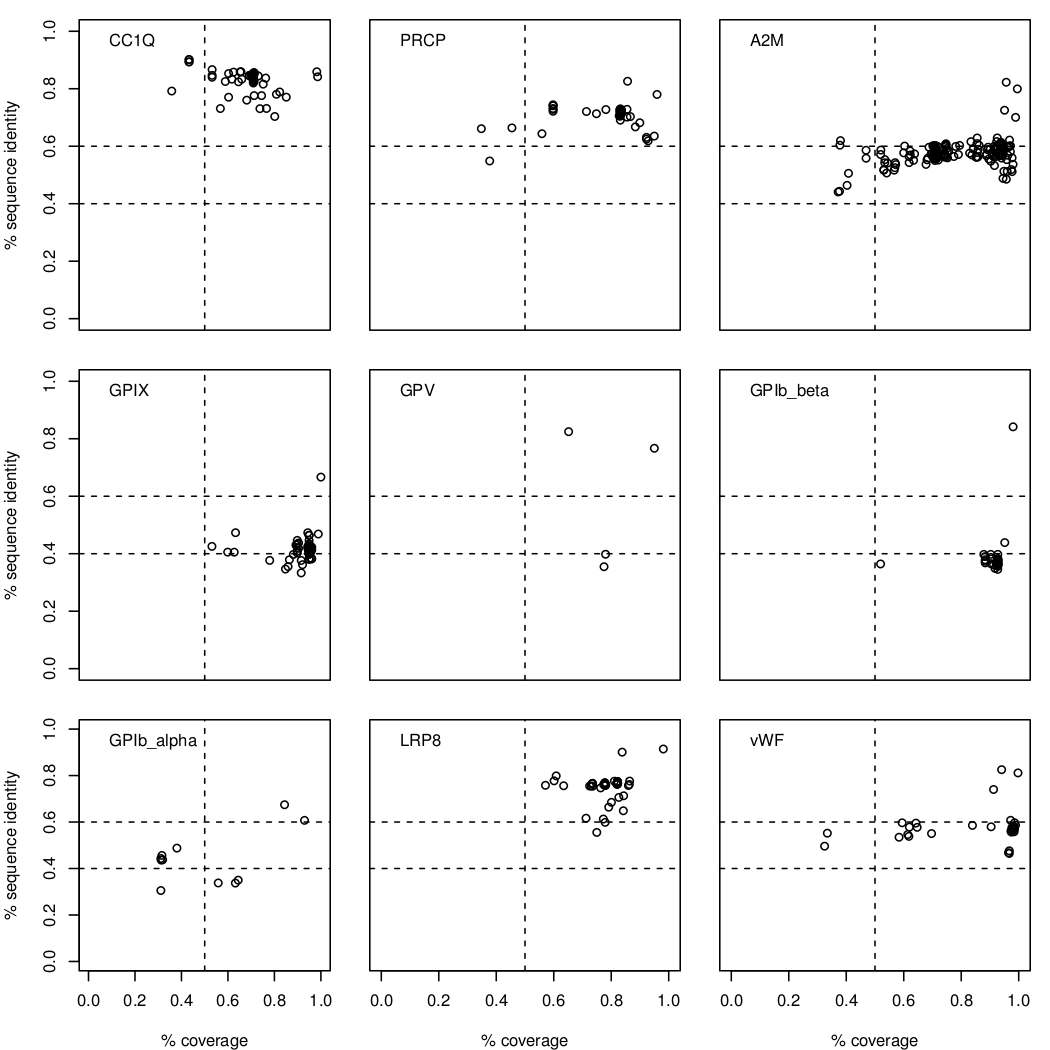
**

Figure S1. Percent sequence identity and coverage for all the filtered BLAST hits. Includes all the elements of the ortholog groups found by searching genomes and transcriptomes across vertebrates that matched the basic orthology assignment criteria (see Methods).

******

Figure S2. Multispecies alignment of GP1balpha. The amino acid sequences were aligned with MAFFT^[[1]](#footnote-1)^ using the Blosum62 score matrix as implemented in Geneious v7.0 (Biomatters Ltd). The absence of the cytoplasmic tail of GP1balpha is notorious in archosaurs (birds and crocodiles) and turtles. Four birds are included in the depiction. Species acronyms are reported in Table S2. TM domain: trans-membrane domain.

******

Figure S3. FastTree [38] phylogeny of the FXI-KLK genes. The duplication that gave rise to KLKB1 seems to have occurred in therian lineage that includes placentals (‘HUMA’: human; ‘BOTA’: cattle) and marsupials (‘MODO’: opossum). Prototheria (‘ORAN’: platypus), as other vertebrates, have a single FXI-KLK gene (other species acronyms in Table S2).


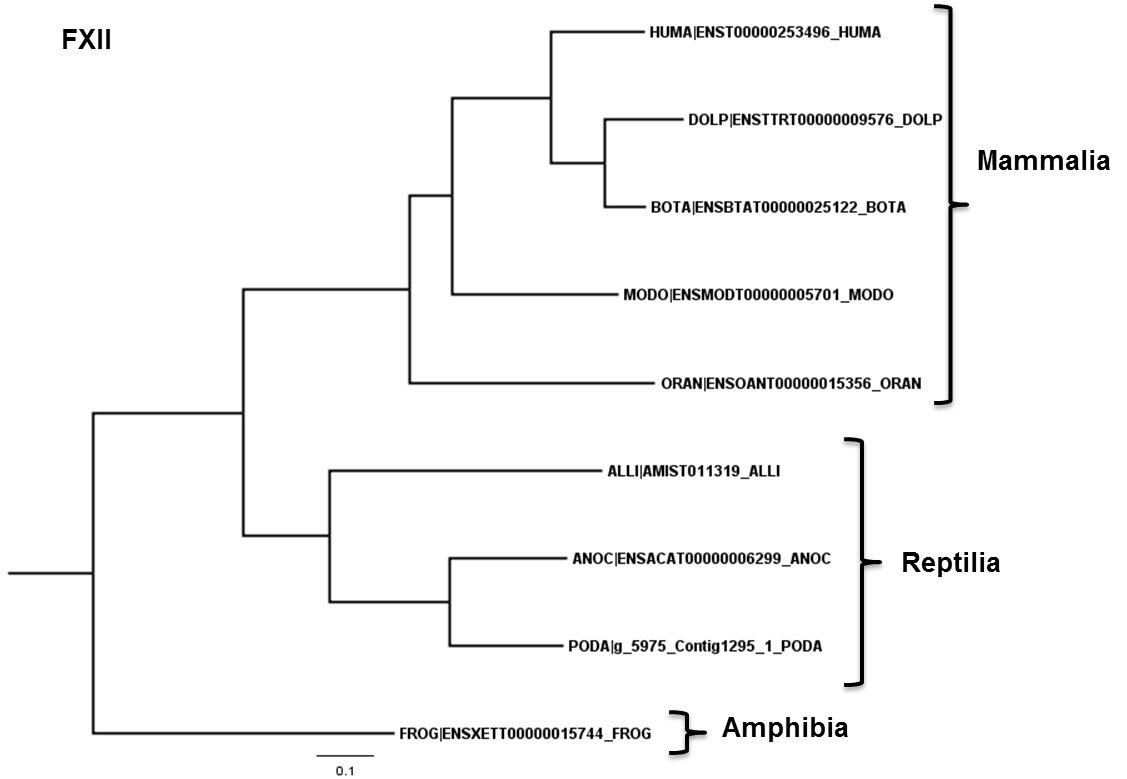


Figure S4. FastTree [38] phylogeny of the detected FXII orthologs, noting its absence in birds.

1. Supplementary Tables

**Table S1.** Protein names and ID according to the KEGG pathway database: coagulation network ko04610 and extracellular matrix receptor interaction ko4512.

| Functional Component | Ensembl IDs | Protein name | Acronym | KEGG ID |
| --- | --- | --- | --- | --- |
| **Subendothelium components** | ENST00000261405 | von Willebrand Factor | vWF | K03900 |
| **Platelet** | ENST00000329125 | Glycoprotein Ib subunit alpha | GPIbα | K06261 |
|  | ENST00000366425 | Glycoprotein Ib subunit beta | GPIbβ | K06262 |
|  | ENST00000323007 | Glycoprotein V | GPV | K06260 |
|  | ENST00000307395 | Glycoprotein IX | GPIX | K06263 |
|  | ENST00000306052 | Low density lipoprotein receptor-related protein 8 | LRP8 | 7804 |
| **Plasma Proteins** | ENST00000311907 | Coagulation Factor II, Thrombin | FII | K01313 |
|  | ENST00000360256 | Coagulation Factor VIII | FVIII | K03899 |
|  | ENST00000218099 | Coagulation Factor IX | FIX | K01321 |
|  | ENST00000375559 | Coagulation Factor X | FX | K01314 |
|  | ENST00000264692 | Coagulation Factor XI | FXI | K01323 |
|  | ENST00000253496 | Coagulation Factor XII | FXII | K01328 |
|  | ENST00000511608 | Plasma Kallikrein | KLK | K03910 |
|  | ENST00000447445 | Kininogen | HK | K03898 |
|  | ENST00000225698 | Complement Component 1 Binding Protein | CC1Q | K15414 |
|  | ENST00000403558 | Serpin G1 | SERPING1 | K04001 |
|  | ENST00000318602 | Alpha-2-macroglobulin | A2M | K03910 |
|  | ENST00000393399 | Prolylcarboxypeptidase or Angiotensinase C or | PRCP | K01285 |
|  | ENST00000205948 | ApoH, beta2glycoprotein I | ApoH | -- |

**Table S2**. Vertebrate species for which genomic data was compiled and the corresponding searched databases.

| Class | Species | Common name | Acronym | Source |
| --- | --- | --- | --- | --- |
| Osteichthyes | *Dario rerio* | Zebrafish | ZFIS | a |
|  | *Takifugo rubripes* | Tiger puffer | FUGU | a |
|  | *Tetraodon nigroviridis* | Green Spotted Puffer | TETR | a |
| Amphibia | *Xenopus tropicalis* | Western clawed frog | FROG | a |
|  | *Ambystoma mexicanum* | Axolotl | MEXI | b |
|  | *Ambystoma tigrinum* | Tiger salamander | TIGR | b |
| Reptilia | *Annolis carolinensis* | Carolina anole | ANOC |  |
|  | *Thamnophis elegans* | Western Terrestrial Garter Snake | SNAKE | d |
|  | *Podarcis sp* | Wall lizards | PODA | e |
|  | *Emuys orbicularis* | European pond turtle | EMYS | f |
|  | *Caretta caretta* | Loggerhead sea turtle | CARET | f |
|  | *Chelonoids nigra* | Galápagos giant tortoise | CHELO | f |
|  | *Chelonia mydas* | Green sea turtle | CHEM | c |
|  | *Pelodiscus sinensis* | Chinese softshell turtle | PELS | c |
|  | *Alligator mississipiensis* | American alligator | ALLI | g |
|  | *Caiman crocodilus* | Spectacled caiman | CAIMA | g |
|  | *Gavialis gangeticus* | Gharial | GAVI | g |
|  | *Crocodylus porosus* | Saltwater crocodile | PORO | g |
| Aves | *Acanthisitta chloris* | Rifleman | ACACH | c |
|  | *Anas platyrhynchos* | Mallard | ANAPL | h |
|  | *Apaloderma vittatum* | Bar-tailed trogon | APAVI | c |
|  | *Aptenodytes forsteri* | Emperor penguin | APTFO | c |
|  | *Balearica regulorum* | Grey crowned crane | BALRE | c |
|  | *Buceros rhinoceros* | Rhinoceros hornbill | BUCRH | c |
|  | *Calypte anna* | Anna's hummingbird | CALAN | c |
|  | *Caprimulgus carolinensis* | Chuck-will's-widow | CAPCA | c |
|  | *Cariama cristata* | Red-legged Seriema | CARCR | c |
|  | *Cathartes aura* | Turkey vulture | CATAU | c |
|  | *Chaetura pelagica* | Chimney swift | CHAPE | c |
|  | *Charadrius vociferus* | Killdeer | CHAVO | c |
|  | *Chlamydotic macquenii* | MacQueen's bustard | CHLMA | c |
|  | *Columba livia* | Rock dove | COLLI | i |
|  | *Colius striatus* | Speckled mousebird | COLST | c |
|  | *Corvus brachyrhynchos* | American crow | CORBR | c |
|  | *Cuculus canorus* | Common cuckoo | CUCCA | c |
|  | *Egretta garzetta* | Little egret | EGRGA | c |
|  | *Eurypyga helias* | Sunbittern | EURHE | c |
|  | *Falco peregrinus* | Peregrine falcon | FALPE | c |
|  | *Fulmarus glacialis* | Northern Fulmar | FULGL | c |
|  | *Gallus gallus* | Red junglefowl | GALGA | j |
|  | *Gavia stellata* | Red-throated loon | GAVST | c |
|  | *Geospiza fortis* | Medium ground finch | GEOFO | c |
|  | *Haliaeetus albicilla* | White-tailed eagle | HALAL | c |
|  | *Leptosomus discolor* | Cuckoo roller | LEPDI | c |
|  | *Manacus vitellinus* | Golden-collared manakin | MANVI | c |
|  | *Meleagris gallopavo* | Wild turkey | MELGA | k |
|  | *Melopsittacus undulatus* | Budgerigar | MELUN | l |
|  | *Merops nubicus* | Northern carmine bee-eater | MERNU | c |
|  | *Mesitornis unicolor* | Brown mesite | MESUN | c |
|  | *Nestor notabilis* | Kea | NESNO | c |
|  | *Nipponia nippon* | Crested ibis | NIPNI | c |
|  | *Ophisthocomus hoazin* | Hoatzin | OPHHO | c |
|  | *Pelecanus crispus* | Dalmatian pelican | PELCR | c |
|  | *Phalacrocorax carbo* | Great cormorant | PHACA | c |
|  | *Phaethon lepturus* | White-tailed tropicbird | PHALE | c |
|  | *Phoenicopterus ruber* | American flamingo | PHORU | c |
|  | *Picoides pubescens* | Downy woodpecker | PICPU | c |
|  | *Podiceps cristatus* | Great crested grebe | PODCR | c |
|  | *Pterocles guturalis* | Yellow-throated sandgrouse | PTEGU | c |
|  | *Pygoscelis adeliae* | Adélie penguin | PYGAD | c |
|  | *Struthio camelus* | Ostrich | STRCA | c |
|  | *Taeniopygia guttata* | Zebra finch | TAEGU | m |
|  | *Tauraco erythrolophus* | Red-crested turaco | TAUER | c |
|  | *Tinamus guttatus* | White-throated tinamou | TINGU | c |
|  | *Tyto alba* | Barn owl | TYTAL | c |
| Mammalia | *Ornithorhynchus anatinus* | Platypus | ORAN | a |
|  | *Monodelphis domestica* | Gray short-tailed opossum | MODO | a |
|  | *Tursiops truncatus* | Dolphin | DOLP | a |
|  | *Bos taurus* | Cattle | BOTA | a |
|  | *Homo sapiens* | Human | HOMO | a |

a -ENSEMBL

b - Ambystoma database v4.0: http://www.ambystoma.org

c - C 2014. Science; Bird Phylogenomics Project - <http://phybirds.genomics.org.cn>

d -Schwartz *et al* 2010; Snake database: http://eco.bcb.iastate.edu/

e - Bird Phylogenomics Project - <http://phybirds.genomics.org.cn>

f - Chiari *et al*. 2012 BMC Biology 10:65

g - St. Johns *et al*. 2012; ICGWGC: International Crocodilian Genomes Working Group Consortium; http://www.crocgenomes.org;

h - Huang *et al.* 2013 Nat Genet.

i - Shapiro *et al*. 2013 Science

j - International Chicken Genome Sequencing Consortium 2004 Nature

k - Dalloul *et al.* 2010 PLoS Biol.

l - Ganapathy *et al.* 2013 GigaScience

m - Warren *et al.* 2010 Nature

1. Katoh, Kazutaka; Kuma, Kei-ichi; Toh, Hiroyuki; Miyata, Takashi (2005). "MAFFT version 5: improvement in accuracy of multiple sequence alignment". Nucleic Acids Research 33 (2): 511–8 [↑](#footnote-ref-1)
